# DeepHP: A New Gastric Mucosa Histopathology Dataset for *Helicobacter pylori* Infection Diagnosis

**DOI:** 10.3390/ijms232314581

**Published:** 2022-11-23

**Authors:** Wanderson Gonçalves e Gonçalves, Marcelo Henrique Paula dos Santos, Leonardo Miranda Brito, Helber Gonzales Almeida Palheta, Fábio Manoel França Lobato, Samia Demachki, Ândrea Ribeiro-dos-Santos, Gilderlanio Santana de Araújo

**Affiliations:** 1 Laboratory of Human and Medical Genetics, Institute of Biological Sciences, Graduate Program of Genetics and Molecular Biology, Federal University of Pará, Belém 66075-110, Brazil; 2 Research Center on Oncology, Graduate Program of Oncology and Medical Science, Federal University of Pará, Belém 66073-000, Brazil; 3 Laboratory of Applied Computing, Engineering and Geoscience Institute, Federal University of Western Pará, Santarém 68040-255, Brazil

**Keywords:** deep learning, *Helicobacter pylori*, computational pathology

## Abstract

Emerging deep learning-based applications in precision medicine include computational histopathological analysis. However, there is a lack of the required training image datasets to generate classification and detection models. This phenomenon occurs mainly due to human factors that make it difficult to obtain well-annotated data. The present study provides a curated public collection of histopathological images (DeepHP) and a convolutional neural network model for diagnosing gastritis. Images from gastric biopsy histopathological exams were used to investigate the performance of the proposed model in detecting gastric mucosa with *Helicobacter pylori* infection. The DeepHP database comprises 394,926 histopathological images, of which 111 K were labeled as *Helicobacter pylori* positive and 283 K were *Helicobacter pylori* negative. We investigated the classification performance of three Convolutional Neural Network architectures. The models were tested and validated with two distinct image sets of 15% (59K patches) chosen randomly. The VGG16 architecture showed the best results with an Area Under the Curve of 0.998%. The results showed that CNN could be used to classify histopathological images from gastric mucosa with marked precision. Our model evidenced high potential and application in the computational pathology field.

## 1. Introduction

Gastric cancer is the fifth most common cancer worldwide and the fourth in deaths caused by cancer in 2020 [1]. *Helicobacter pylori* (HP) infection is the main risk factor accounting for around 89% of the distal gastric cancer cases around the world [2]. The HP is extremely adapted to the human gastric mucosa. However, disordered HP proliferation induces inflammation in the gastric mucosa, which can sequentially result in gastric cancer development [3]. It can bind to epithelial cells and prevent the immune response to cancer. HP has a high prevalence worldwide, of almost 50% [4], and 1–3% of the cases of *H. pylori* infection progress to gastric cancer [5].

Endoscopy is the main procedure for assessing HP infection and gastric cancer, followed by an histopathological biopsy analysis [6]. The histopathological analysis allows for identifying HP ubiquity and morphological alterations in the gastric mucosa. A gastric biopsy analysis is a highly time-consuming task. It can be affected by biotechnological factors such as staining techniques, errors in gathering biopsy sites, and also the pathologists’ subjectivity/experience [7,8].

Intelligent computational models are promising in the medical domain in terms of assisting clinical decisions [9]. Few studies have been proposed for gastric-related diseases [10]. Currently, deep learning methods, such as Convolutional Neural Networks (CNNs), are the cutting-edge in research applications for gastric cancer analysis. Moreover, one of the main problems in deep learning applications is the scarcity of large image collections for training to generate classification, segmentation, or detection models, mainly due to complexities in obtaining well-annotated data [10,11]. The absence of high-quality datasets impairs the development of new and optimized tools for the biopsy image analysis of gastric mucosa diseases, making it nearly impossible to develop comparative studies between computational models. In addition, we previously identified several issues regarding model reproducibility caused by a lack of experimental setup information, such as uninformed network hyper-parameters, computational cost, and the availability of private or public image collections [10,12].

Our main contributions are summarized as follows. First, we provide DeepHP, a publicly curated dataset of histopathological images for HP classification for diagnostic studies. This dataset was built by following a detailed protocol to provide trustworthiness in the subjacency analysis. Among the 394,926 patched images, 283,921 were identified as negative for HP infection and 111,005 as positive for HP infection. Secondly, we performed extensive experiments and adopted transfer learning and fine-tuning strategies to analyze the performance of three CNN models in classifying HP infection with our dataset. The best CNN model showed an Area Under the Receiver Operating Characteristic (ROC) Curve (AUC), F1-Score, and Mathews Correlation Coefficient (MCC) of 0.99, 0.96, and 0.95, respectively. The transfer learning and fine-tuning approach to histopathological images showed promising results and can be used for advancements in patient-level classification for diagnostic support.

### Related Works

There are few research studies concerning the application of deep learning for classification, detection, or segmentation tasks to detect gastritis or HP infection. The challenge is to obtain reliable, robust, and curated histopathological image collections for training, testing, and validating CNN models. Beyond that, deep learning methods have shown encouraging results in the classification and detection of gastritis [13,14,15]. Those classification and detection models have been built using X-ray images, which are not invasive, and the applications showed good performance. In some ways, the automated analysis of endoscopic images were superior to that of endoscopists in the classification task [13,14,15].

Endoscopy images are widely used to detect HP infection with pre-trained networks (e.g., GoogleNet, ResNet-50, or Inception v3) based on transfer learning and fine-tuning strategies [16,17,18,19,20,21]. Interestingly, Nakashima et al. [22] trained a distinct CNN to classify endoscopy video frames between HP positive and negative samples.

Martin et al. [23] trained the HALO-AI software to classify gastric images with HP–related gastritis, reactive gastropathy, and histopathologically normal gastric mucosa. Their dataset consisted of 300 images of classic cases that best represented the desired pathology, divided into 100 for each category, and 106 other examples of non-classical gastric biopsies. Around 70% of the 300 images were used for training, and the other 30%, plus the 106 non-classical case images, were used for testing.

Despite several works dedicated to analyzing gastroenterological issues, we did not find public links for image datasets, including the previous research studies discussed in this section. Thus, our work contrasts by providing enough information and data to improve research replicability. The dataset with curated histopathological images labeled HP positive/negative is publicly available.

The remainder of this paper is organized as follows. Results are presented and discussed in Section 2. In Section 3, the material and methods are presented. A critical comparison of the obtained results with state-of-the-art is given in Section 2.3. Finally, the conclusions are presented in Section 4.

## 2. Results and Discussion

In this Section, we present and discuss the results of both generating the DeepHP and the transfer learning experiments.

### 2.1. The Working DeepHP Dataset

Deep learning has been applied to gastroenterological problems on private image collections from distinct research centers. Consequently, we did not find publicly available datasets, impairing the experiments’ transparency and reproducibility [10]. The growth of deep learning applications requires a large volume of reliable data to fill the gap in medical applications such as gastroscopy, colonoscopy, and digital pathology to properly train deep learning networks [24].

Our data were obtained from a pathological anatomy archive with diagnoses already established for each slide. The preprocessing strategy was performed to separate tissue and tissue-free images in patches. The filtering strategy resulted in a dataset with 283,921 patches diagnosed as *H. pylori* negative and 111,005 patches diagnosed as *H. pylori* positive. The agreement between the raters produced a $\hat{\kappa }$ coefficient of close to 0.95, which was evaluated as almost perfect. The overall accuracy of the filter defined as Rater A was 0.98, while Rater B achieved 0.97.

This dataset was used in the pre-trained CNN models to assess image classification, i.e., whether they were HP positive or negative. In our experiments, 70% of gastric slices (both *H. pylori* positive and *H. pylori* negative) were randomly selected for CNN training, 15% of slices were used for validation, and 15% were used for the testing steps.

### 2.2. Transfer Learning and Fine-Tuning Results

There is still a great deal of discussion about the number of images needed for deep learning. However, within the learning scenario, transfer learning is the most popular due to the versatility of using external data [25]. Thus, with this image collection, we used transfer learning techniques and fine-tuning to evaluate the performance of the following distinct CNN architectures: VGG16, InceptionV3, and ResNet50.

In general, studies that address deep learning applications in image classification, detection, or segmentation usually use accuracy as the predominant metric [10]. In addition, we evaluate CNN models with the F1-Score and MCC for an informative and truthful score in evaluating binary classifications [26]. The F1 score is an evaluation metric that considers recall and precision in its formulation; it is a more balanced metric than the unit elements of the confusion matrix. However, the result can be biased because it does not include the true negatives. Thus, the F1 score is easily influenced by the class labeled as positive [27]. In contrast, the MCC considers all cells from the confusion matrix and will only perform well if the classifications of the positive and negative classes are also equally good [26]. Therefore, the interpretation of these metrics depends on the study domain. The F1-Score will address problems that give preference to a specific positive class; however, if the cost of low precision and low recall is unknown, the MCC is preferable to the F1-score. We also performed AUC analyses to verify the sensitivity and specificity of the models.

The performance of each model trained without transfer learning is shown in Table 1. The VGG16 has significantly better performance than the InceptionV3 and ResNet50 models, according to MCC ≥ 0.88. Additionally, we evaluated the models by implementing the fine-tuning approach with the test data. Results for the fine-tuning performance are shown in Table 2. VGG16 outperforms InceptionV3 and ResNet50 after fine-tuning. The results in Table 1 and Table 2 make clear the effectiveness of the fine-tuning approach; we observed an improvement in the pre-trained VGG16 and InceptionV3 models with the fine-tuning approach, while the ResNet50 model showed a decreased performance, which may be due to the new data domain or the choice of unfrozen blocks during the fine-tuning process.

VGG may perform equivalently in similar independent datasets. For example, HALO-AI implements a fully convolutional version of the VGG architecture [23]. Indeed, model performance could be increased with hyperparameter optimization. Although the parameters for VGG16 are higher in this work, we still see that there is a good performance in the other models (ResNet50 and InceptionV3). The complexity of the models, the pre-training set, the adjusted hyperparameters, and the dataset configuration itself are factors that greatly influence the final result. Consequently, changing these factors can easily affect the performance of the CNN models.

**Figure 1:**
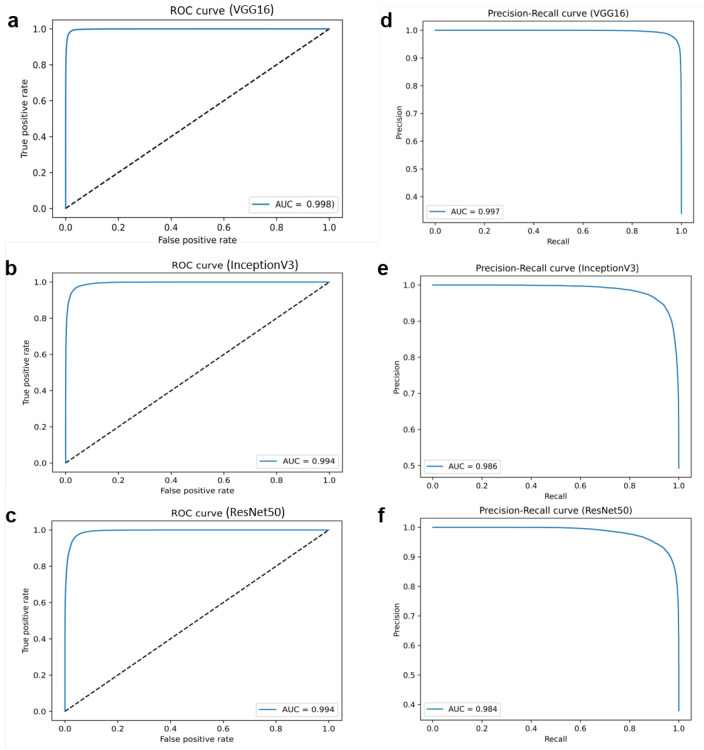
Results of Receiver Operating Curves (ROC) and AUC for VGG16 (**a**), InceptionV3 (**b**), and ResNet50 (**c**). Results of Precision–Recall curve (PRC) and AUC for VGG16 (**d**), InceptionV3 (**e**), and ResNet50 (**f**).

Additionally, we used the Receiver Operating Characteristic Curve (ROC curve) to determine the accuracy of the CNN decision. The ROC curve is derived from the graph of sensitivity against specificity, for which we calculated the AUC. No cut-off values (values that differentiate between positive and negative results) were used, and the ROC curve was obtained when plotting the values. Values ranged from 0 to 1, with values close to 1 indicating a high classification accuracy. The results of the ROC curves and the respective AUCs are shown in Figure 1. The ROC graph plots the false positive rate (FPR) against the true positive rate (TPR). Similarly, the Precision–Recall curve (PRC) is an alternative to the ROC curve for tasks with large deviations in the class distribution. Furthermore, PR curves can expose differences between algorithms that are not apparent in the ROC [28]. The results of the Precision–Recall curves and the respective AUCs are shown in Figure 1 which shows VGG16 has good performance (AUC = 0.997).

However, common knowledge in the scientific community points to unbalanced data as those that correspond to a dataset with significant and even extreme imbalances, which is not the case in this work. These imbalances are commonly attributed to class ratios in the order of 100:1, 1000:1, and 10,000:1, where in each case one class severely represents another [29,30].

### 2.3. Related Studies and Analysis

Our study surpassed others involving endoscopy imaging [17,18]. Deep learning analyses of HP infection by upper gastrointestinal endoscopy images showed a sensitivity and specificity of 86.7% and 86.7%, respectively, and the AUC equals 0.956 [18]. In [17], which implements GoogleNet fine-tuning to diagnose HP infection, provided a promising diagnostic model with an Area Under the Curve 0.96 and 0.95 using Bright Blue (BLI-bright) and linked color image laser image data.

In Martin et al. [23], the authors used HALO-AI image analysis on tissue with HP-related gastritis, reactive gastropathy, and gastric mucosa. Their dataset consisted of 300–100 images for each category and 106 other examples of non-classical gastric biopsies. Around 70% of the 300 images were used for training, and the other 30%, plus the 106 non-classical case images, were used for testing. The AUCs were 91.9% for normal, 100% for *H. pylori*, and 94.0% for reactive gastropathy. The sensitivity and specificity were as follows: normal (73.7%, 79.6%), *H. pylori* (95.7%, 100%), reactive gastropathy (100%, 62.5%) in 130 additional gastric biopsies. The HALO-AI software assigned color labels to the test biopsies, which correspond to the area of tissue assigned to a diagnosis by the DL algorithm, called the area distribution. For the results presented above, there was an area distribution cutoff of 40%.

The mentioned study used established software to train and test its dataset. This software allows the analyst to contour the areas of interest on the whole slide. Pathologists make this markup in the software itself, and this information is provided as input for network training. Slides were taken at ×5.5 magnification. In addition, the authors used a cut-off in their evaluation metrics.

In comparison to our study, there are significant differences in the dataset acquisition method and image magnification. We used image patches, pre-trained CNN models with classification layer adjustments, and the application of fine-tuning with ×20 magnification. We obtained a classification result for HP infection and without HP infection, without cutoffs, with an AUC of 0.998. This result represents a discriminative power of identifying inflammatory profiles associated with HP infection at the level of image patches. The use of cutoffs can further enhance the ability of classification.

In recent years, the regular use of AI-based medical devices, including CNN applications, has received much attention. The FDA has already approved two medical devices, the IDX-DR for image analysis for the detection of diabetic retinopathy and one called Viz.AI, which analyzes images for indicators of stroke [31]. The use of these devices is becoming a reality in the medical context. That is why regulatory agencies are concerned about methodologies and these applications, which can increase the reliability and effectiveness of medical assistance. There are still discussions about how these artificial intelligence algorithms create their problem-solving rules. These applications become so complex that they hide what happens in multiple layers, called “black-box” algorithms. In Europe, the European Union’s General Data Protection Regulation (GDPR) implies that language needs to be fully explained; therefore, an algorithm cannot be used without revealing its “black-box”. However, given the robust results of software AI applications in the medical field, such as in the detection of melanoma, where CNN obtained results superior to a group of human experts [32], the FDA did not establish such a barrier and published a discussion paper that proposes a regulatory framework for software modifications based on artificial intelligence in computer-assisted diagnosis [31,33].

There are some limitations in our study that should be pointed out. First, we used a few examples to generate the database. This limitation makes the CNN constrained to the generated dataset. More images can help to provide a more significant dataset variability and, consequently, better generalization of the model. This limitation can be solved in future studies by including more slides. Obtaining health data is always a sensitive topic, with problems ranging from ethical and patient privacy issues to the selection of appropriate equipment for image acquisition. Second, training and testing data collection was performed at a single center. The use of data from other research/diagnostic centers could improve sample heterogeneity for model validation and generalization. However, as mentioned earlier, data acquisition in healthcare can be a difficult and time-consuming process. We seek cooperative agreements with other research centers to overcome this gap.

## 3. Material and Methods

This section is divided according to our outcomes, the DeepHP dataset, and the experiments with CNNs.

### 3.1. DeepHP Dataset

This subsection encompasses data acquisition, pre-processing, filtering, and validation.

#### 3.1.1. Images Collection

The images used to build the dataset were collected from gastric biopsies stained with hematoxylin-eosin (H&E) using histopathological Whole Slide Imaging (WSI). All digital images are RGB, 0.16 μm per pixel, scanned with a ZEISS Microscope—Axio—imager.M2, ×20 magnification. Histopathological samples are anonymous images of human gastric mucosa embedded in paraffin fixed in formalin and stained with H&E from the pathological anatomy archive of Hospital Universitário João de Barros Barreto, Federal University of Pará (Belém, Pará, Brazil).

The original dataset consists of 13,921 images generated from 19 histopathological WSI, 14 gastric mucosae without morphological changes (9926 negative HP samples images), and five with HP infection (3995 positive sample images). Each image has dimensions of 2776 × 2080 pixels. The original DeepHP image collection can be requested by following the instructions in the DeepHP section at https://www.lghm.com.br/datasets (accessed on 22 November 2022).

#### 3.1.2. Image Pre-Processing

**Figure 2:**
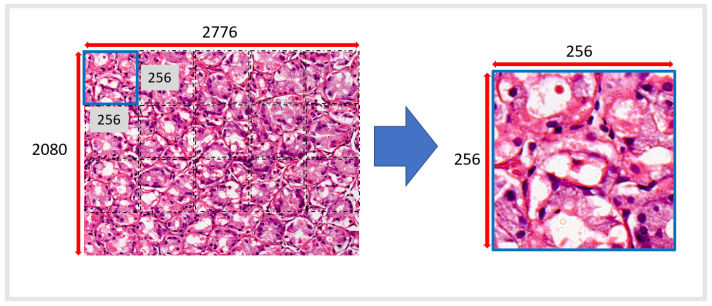
A random example of the original H&E image (2776 × 2080 pixels) and one patch sized to 256 × 256 pixels.

Each image (2776 × 2080 pixels) was split into smaller non-overlapped image patches of 256 × 256 pixels using grid sampling (see a random sample in Figure 2). After this process, our dataset comprised 1,113,680 patches sized to 256 × 256 pixels (794,080 negative *H. pylori* and 319,600 positive *H. pylori*).

During the capture process of biopsy images, we observed the absence of gastric tissue in some images after automatic patch cropping. We implemented a pre-processing strategy to detect the tissue and the bottom of the slide. The goal was to keep samples filled with at least 50% of gastric tissue in the patch. The images with less than 50% of observable tissue in the patch were moved out of the working dataset. The approach is described in Algorithm 1.    
**Algorithm 1:** Image selection algorithm.
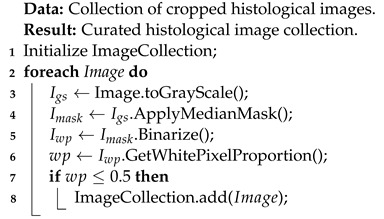


**Figure 3:**
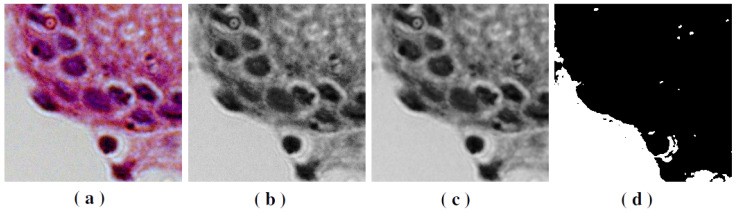
Example image patch filtering process. (**a**) An original patch (256 × 256 pixels); (**b**) patch after grayscale transformation; (**c**) smoothing filter for noise correction; (**d**) gray-scale patch is binarized from the 0–255 spectrum to a 0–1 spectrum.

The patches are RGB figures, which were transformed into grayscale. This step is crucial to increasing the speed of image operations. Then, we apply a median mask to reduce image noise. After this smoothing, the patches were linearized. Finally, the proportion of white pixels is measured, which corresponds to the image background. If this proportion is more than 50% (our percentage threshold), the patch is removed from the working database; otherwise, it is retained. This process can be seen in Figure 3.

For filter validation, the patches were sampled according to Expression (1) as follows, with a 95% confidence interval:

(1) $$ss=\frac{\frac{z^{2}p\left(1-p\right)}{e^{2}}}{1+(\frac{z^{2}p\left(1-p\right)}{e^{2}N})},$$

where ss is a sample size, *N* is the population size, *e* corresponds to the margin of error (percentage in decimal format), *p* represents the expected proportion and *z* is the *z*-score, which is the number of standard deviations between a given ratio and the mean.

Subsequently, 400 samples were selected randomly for each of the 19 patch folders. Image binarization limits and white ratio were applied, as shown above. Two independent raters defined the filter thresholds in the sample data (see Table 3). Each rater received a copy of the same sample set, then the values were assessed through subjective inspection.

Afterward, the threshold average was applied to the original sample data patch, and each inspector evaluated the result independently. The result of the average thresholds between the evaluators is presented in Section 2.1.

#### 3.1.3. Image Validation

The statistical concordance (with or without tissue) was made using the Kappa coefficient ($\hat{\kappa }$). The evaluation of the automatic fragment separation filter was carried out using the confusion matrix and global accuracy. The global accuracy was calculated according to the ratio between the sum of true positives and true negatives (TP+TN) and the sum of total positives and negatives (P+N), as described in Equation (2).

(2) $$accuracy=\frac{TP+TN}{P+N}$$

Cohen begins by defining a confusion matrix *k* by *k* and calculates the $\hat{\kappa }$ coefficient according to Equation (3) [34]):

(3) $$\hat{\kappa }=\frac{\hat{p_{o}}-\hat{p_{e}}}{1-\hat{p_{e}}}$$

Being the overall proportion of observed agreement ($\hat{p_{o}}$):,

(4) $$\hat{p_{o}}=\sum_{i=1}^{k}\frac{p_{ii}}{n},$$

and the overall proportion of chance-expected agreement ($\hat{p_{e}}$):

(5) $$\hat{p_{e}}=\sum_{i=1}^{k}\frac{p_{i.}p_{.i}}{n^{2}}.$$

where *k* represents the evaluation categories, $p_{.i}$ represents the number of samples evaluated by rater Y in category *i*, $p_{i.}$ represents the number of sample elements evaluated by rater X in category *i* and *n* represents the total samples evaluated.

The Kappa coefficient (κ) is suitable for a better model/annotation process assessment since it takes into account all the elements of the confusion matrix, different from the overall accuracy that uses only the main diagonal [35].

In the present work, the Kappa coefficient is compared to the indices proposed by Landis and Koch, 1977 [36], which characterize different ranges of values for kappa concerning the degree of agreement. The indices are shown in Table 4.

### 3.2. Convolutional Neural Network

Convolutional Neural Networks (CNNs) are bio-inspired neural networks that learn through the spatial structure of the images and the dependence between pixels close to each other [37]. Its layers are divided into input, feature extraction, activation, classification, and output layers.

**Figure 4:**
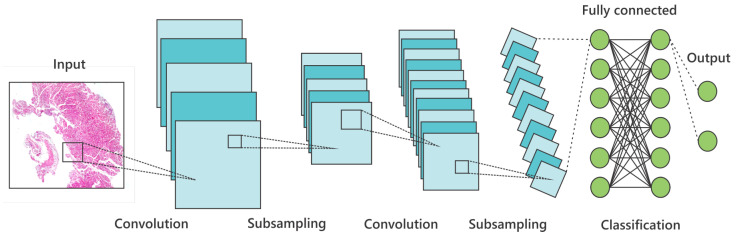
Architecture schema of a standard Convolution Neural Network.

The CNN was implemented as a multilayer architecture based on artificial neural networks (ARNs) and the backpropagation algorithm. Computational costs have limited the development of this machine learning subfield, the amount of data available, and restrictions of classical techniques [38]. Figure 4 illustrates the structure of a CNN. The first layer in the process is the input layer. This step is responsible for placing the original images of the problem to be solved in the network. The next step is to insert feature extraction layers, which correspond to Convolutional and Grouping Layers (or subsampling), using kernels or spatial filters, extracting important features, and removing those not crucial to the process.

The activation function performs nonlinear transformation in the input data, which facilitates the learning and performance of more complex tasks. Each layer becomes an expert in identifying essential image features, such as colors, outlines, curves, formats, and others that depend on the application domain.

The convolutional kernel values are learned by the network in the training phase [39]. This process generates the known activation maps produced by each layer and assists in the feature extraction process. Since pool filters have no defined values, they are used as a logical operation to reduce the network’s processing load. These filters produce another image with a smaller dimension than the previous one, which can be the extraction of the maximum, medium, or minimum value of a pixel within the filter that will be applied to the image.

The image’s dimensions are reduced within the network. The output image is transformed into a 1D characteristic vector, a flat 1D layer at the end of the convolutional and pooling layers application. This long input data vector passes through the artificial neural network, usually a “Multilayer Perceptron” (MLP), to perform the feature classification.

In this way, a CNN can adjust the image dataset by reducing parameters and reusing weights, enabling the successful capture of the spatial and temporal dependencies of the image by applying relevant filters [40]. Based on the CNN model, we investigated the performance of the following three main CNN architectures: VGG16, InceptionV3, and ResNet50.

- VGG16: The first work that popularized CNNs in computer vision is VGG-Nets [41], which directly incorporated classic CNN architectures, and won first and second places in the location and ranking tasks in the ImageNet Challenge 2014, respectively. We opted for the VGG network with 16 weight layers (VGG16), namely thirteen convolutional layers and three fully-connected layers with a final softmax classifier. The convolution operations on the thirteen layers were performed using a 3 × 3 pixel dimension kernel, with the *W* and *b* parameters being slipped over the × pixels of each image resulting in a *y* output (Equation (6)). Kernel offset is pass-dependent and can be pixel-by-pixel or can skip multiple pixels. Convolutional layers act as feature extractors from the input images. The deeper the layers, the more specific the details that are extracted, and the earlier the layers, the more general the features that are extracted. The activation function of each convolutional layer is the Linear Rectified Unit (ReLU) for removing the linearity factor. The convolution operations results, also called feature maps, receive a pooling layer to reduce dimensionality, and in this case, max-pooling is applied in a 2 × 2 pixel window, with step 2. Finally, three layers that are Fully-Connected (FC) follow a stack of convolutional layers, two layers have 4096 neurons, and the third performs 1000 classifications and therefore contains 1000 neurons. The final layer receives the soft-max function [42].
  (6) y=f(Wx+b)
- INCEPTION V3: Proposed by Szegedy et al. in 2015, InceptionV3 uses initial blocks based on a typical CNN architecture [41]. This architecture achieved good performance with relatively low computational cost, which was enhanced by the orderly addition of RMSProp, label smoothing, 7 × 7 factoring, and BN auxiliary layers in the InceptionV2 network [43]. The network includes eleven Inception modules of five types, concatenated to obtain maximum resource extraction. Each module is branched and applies different kernel sizes (1 × 1, 3 × 3, 5 × 5, and 7 × 7). These filters extract and concatenate different scales from feature maps. In the following step, 1 × 1 convolutions are applied for dimensionality reduction before computationally more expensive 3 × 3 and 5 × 5 convolutions. The factoring strategy of 5 × 5, and 7 × 7 convolutions into smaller convolutions (3 × 3) or asymmetric convolutions (1 × 7, 7 × 1) is applied to reduce the number of parameters [44]. In summary, InceptionV3 uses symmetrical and asymmetrical components, including convolutions, average clusters, maximum clusters, concatenations, dropouts, and fully connected layers. Batch normalization is used extensively throughout the applied mode to trigger inputs.
- RESNET 50: One of the problems that occur with very deep networks is the well-known vanishing gradient. This problem occurs when the loss function gradients tend to zero after numerous partial derivatives in each training iteration are found. This process prevents the weights from being upgraded, thus, stopping the learning process. A Deep Residual Network (ResNet) uses a two-layer convolutional building block with an identity connection jumping over them [45]. It is similar to networks with convolution, pooling, activation, and fully connected layers stacked on top of each other. What makes it a residual network is the identity connection between the layers. In this way, the input of the model’s first layer then becomes the output of the last layer, the network must be able to predict any function it has previously learned with the input added to it. Gradients can flow directly through jump-back connections from the later layers to initial filters [45]. A specific ResNet network is instantiated by stacking these building blocks at the desired depth. ResNet-50 is a convolutional neural network that has 50 layers. The difference in accuracy between ResNet-18 and ResNet-50 was notable in a performance comparison [46]. For ResNet networks with depths of greater than 50, the increase in performance was moderate.

### 3.3. Training Strategy of CNN Models

**Figure 5:**
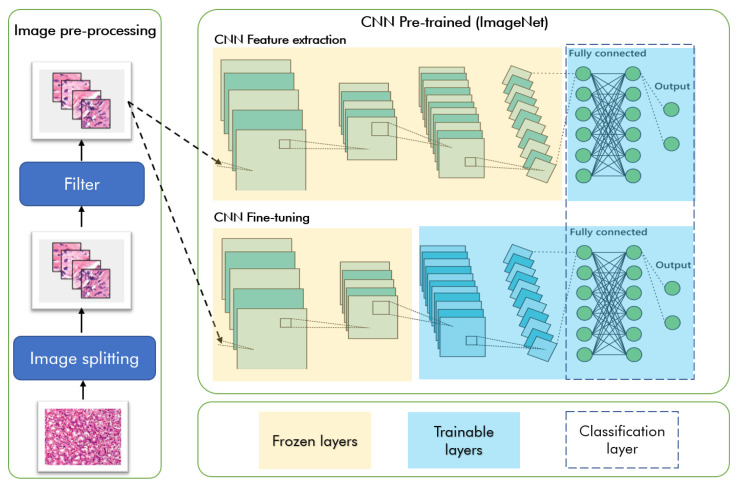
Overview of transfer learning approach. The frozen convolutional layers are highlighted in yellow, and the trainable layers of the models are highlighted in blue.

In this paper, we used transfer learning and fine-tuning approaches (see Figure 5). Transfer learning is characterized by using a trained model in one domain as a starting point for another domain’s training phase, reducing the amount of data required for achieving acceptable results, consequently reducing the processing time [47]. The trained model provides a structure that previously acquired knowledge and can be used to solve new problems, in contrast to the approaches of classical machine learning algorithms [48,49].

The idea behind transfer learning is that if the model is trained on a large enough dataset, then it is likely that general characteristics common to visual systems could be learned. Therefore, it is possible to take advantage of these generated attribute maps to train models without constructing a model from scratch.

In this study, we investigated the performance of pre-trained VGG16, INCEPTION V3, and RESNET 50 models. All models are trained on ImageNet, a large dataset with more than 14 million annotated images and 1000 classes. These CNN architectures are widely addressed in several works involving gastric tissue imaging [10].

DeepHP was randomly divided into the training, validation, and test set. The training dataset was used for CNN training and for the adjustment of hyper-parameters. The validation set was employed to evaluate the CNN models during training and to make adjustments to the model. The test set was used to analyze the model’s performance through evaluation metrics (see Section 3.4) and verify the generalization of the network.

For feature extraction, we added a fully-connected classifier at the top. The pre-trained model is “frozen”, and only the classifier weights are updated during training. In this case, the convolutional base extracted all the resources associated with each image. We only train a classifier that determines the image class with that set of extracted features.

We used a grid search approach on the classifier parameters and evaluated it using 5-fold cross-validation for 20 epochs on a sample with 10% of the dataset. The parameters used in the grid search were the number of fully connected neurons, activation functions, number of layers, dropout, optimizer, and learning rate. For each epoch, 32 samples are randomly selected by using ADA and SGD with 0.01, 0.001, and 0.0001 learning rates to update the network weights. One epoch is over until the whole dataset is used. The sigmoid function was adopted to classify samples, and all networks added one or two full connect layers before the sigmoid layer. All training data were augmented by performing rotation transformation, zooming, horizontal flip, vertical flip, and scale normalization.

After evaluating the training results, we discard the less accurate models and re-apply the above methodology to the total dataset. We select the best combination for each model and then fine-tune it. For fine-tuning, we unlocked part of the convolutional layers. Thus, their weights are adjusted so that the model learns high-level features specific to the dataset. The parameters in the convolutional layers do not need to be changed. However, it is necessary to define how many convolutional layers will be unfrozen. We include an accuracy checkpoint as a monitor to save the most accurate weight set.

We develop our applications in Python, with the Application Programming Interface (API) Keras, working with TensorFlow. Keras is primarily focused on ease of use, modulation, and extension and has well-known models of famous high-performance networks, so it is a powerful tool for transfer learning tasks. We train our networks on a computer with an Intel Core i9 3.7/5.3 GHz processor, 16 GB RAM, 2 TB SATA HDD, 250 GB SSD, and NVIDIA RTX 3070 8 GB. For the total data, we use a computer with an AMD Threadripper 3960X 3.8/4.5 GHz processor, 128 GB RAM—8 × 16 GB, 10 TB SATA HDD, 250 GB SSD, NVIDIA RTX 3090 24 GB.

### 3.4. Evaluation Methods for CNN Models

For CNN model evaluation, we measured the Receiver Operating Characteristic Curve (ROC curve) and the Area Under Curve (AUC), the F1-score defined in Equation (7), and the Matthews correlation coefficient defined in Equation (10).

Based on the ground truth, the F1-score can be calculated as follows:

(7) $$F1=\frac{2*Precision*Recall}{Precision+Recall}=\frac{2*TP}{2*TP+FP+FN}$$

where precision can be calculated by:

(8) $$Precision=\frac{TP}{TP+FP}$$

and recall by:

(9) $$Recall=\frac{TP}{TP+FN}$$

The Mathews correlation coefficient was also used as an evaluation metric. The MCC was first introduced by B.W. Mathews to predict the secondary protein structure [50] and can be calculated using the equation provided below:

(10) $$MCC=\frac{TP*TN-FP*FN}{\sqrt{\left(TP+FP)(TP+FN)(TN+FP)(TN+FN\right)}}$$

## 4. Conclusions

Precision medicine is attaining attention. Deep learning-based applications are providing advancements in patient-level diagnostic support. Some diseases in which the diagnosis depends on a subjectivity analysis, such as histopathological exams, benefit from decision support systems using deep learning approaches. However, there are some challenges to fully implementing reliable decision support systems in this application domain. First, deep learning requires a large volume of data to achieve reliable results. Second, and no less critical, several works that use deep learning in the medical domain are not fully described or provide the datasets, impairing the experiments’ transparency and reproducibility.

Aiming to fill these gaps, we present DeepHP in this work, which is a curated public collection of histopathological images for detecting gastric mucosa with *Helicobacter pylori* infection. Moreover, we tested three deep learning architectures, namely VGG16, InceptionV3, and ResNet50. We conducted extensive computational experiments to test a transfer learning strategies.

Our results conclude that CNN models can detect HP infections and inflammatory profiles in gastric biopsies, providing adequate support for clinical diagnosis, regardless of whether physicians or specialists use the system. The use of accurate CNN models can help reduce the workload and highly laborious tasks of pathologists. In the future, we plan to use image collections with different staining methods and set a cutoff for classifying entire images rather than just patches and advancing parameter adjustment and data augmentation to improve these CNN architectures.

The future research possibilities are broad. Our dataset can be expanded and used by other research groups, considering that it is publicly available. The trained models can be used as a basis for other medical applications. Other CNN models can be explored. Finally, explainable AI methods can be investigated to attend to the FDA and other regulatory agencies’ requirements.

## Figures and Tables

**Table 1:** An overview of the evaluation metric results was obtained for the VGG16, InceptionV3, and ResNet50 models using the transfer learning strategy.

Model	Accuracy	Precision	Recall	Specificity	F1 Score	MCC
**VGG16**	0.95	0.93	0.90	0.97	0.91	0.88
**InceptionV3**	0.94	0.90	0.86	0.96	0.87	0.84
**ResNet50**	0.95	0.85	0.98	0.93	0.91	0.87

**Table 2:** An overview of evaluation metric results was obtained for VGG16, InceptionV3, and ResNet50 using the fine-tuning approach.

Model	Accuracy	Precision	Recall	Specificity	F1 Score	MCC
**VGG16**	**0.98**	**0.94**	**0.99**	**0.98**	**0.96**	**0.95**
**InceptionV3**	0.96	0.88	0.98	0.95	0.92	0.90
**ResNet50**	0.94	0.82	0.99	0.91	0.89	0.86

**Table 3:** Thresholds used in the separation filter for the sample data.

Slide	Rater X Threshold	Rater Y Threshold	Average Threshold
1	196	210	203
2	186	200	193
3	199	215	207
4	176	193	184.5
5	186	195	190.5
6	161	195	178
7	217	235	226
8	204	230	217
9	196	220	208
10	194	205	199.5
11	217	240	228.5
12	204	235	219.5
13	178	205	191.5
14	178	195	186.5
15	204	235	219.5
16	153	190	171.5
17	178	205	191.5
18	191	200	195.5
19	196	220	208

**Table 4:** Classification quality based on the Kappa coefficient values.

Value of Kappa	Level of Agreement
<0.00	Poor
0.00–0.20	Slight
0.21–0.40	Fair
0.41–0.60	Moderate
0.61–0.80	Substantial
0.81–1.00	Almost Perfect
